# Characterization and Comparative Expression Profiling of Browning Response in *Medinilla formosana* after Cutting

**DOI:** 10.3389/fpls.2016.01897

**Published:** 2016-12-22

**Authors:** Yan Wang, Yiting Wang, Kunfeng Li, Xijiao Song, Jianping Chen

**Affiliations:** ^1^State Key Laboratory Breeding Base for Zhejiang Sustainable Pest and Disease Control, Ministry of Agriculture Key Laboratory of Biotechnology in Plant Protection, Institute of Virology and Biotechnology, Zhejiang Academy of Agricultural SciencesHangzhou, China; ^2^Agriculture Experiment Station, Zhejiang UniversityHangzhou, China

**Keywords:** transcriptome, high-throughput sequencing, browning, tissue culture, *Medinilla*, cutting, gene expression profiling

## Abstract

Plant browning is a recalcitrant problem for *in vitro* culture and often leads to poor growth of explants and even failure of tissue culture. However, the molecular mechanisms underlying browning-induced physiological processes remain unclear. *Medinilla* is considered one of the most difficult genera for tissue culture owning to its severe browning. In the present study, intact aseptic plantlets of *Medinilla formosana* Hayata previously obtained by ovary culture, were used to explore the characteristics and molecular mechanism of the browning response. Successive morphological and anatomical observations after cutting showed that the browning of *M. formosana* was not lethal but adaptive. *De novo* transcriptome and digital gene expression (DGE) profiling using Illumina high-throughput sequencing were then used to explore molecular regulation after cutting. About 7.5 million tags of *de novo* transcriptome were obtained and 58,073 unigenes were assembled and annotated. A total of 6,431 differentially expressed genes (DEGs) at three stages after cutting were identified, and the expression patterns of these browning-related genes were clustered and analyzed. A number of putative DEGs involved in signal transduction and secondary metabolism were particularly studied and the potential roles of these cutting-responsive mRNAs in plant defense to diverse abiotic stresses are discussed. The DGE profiling data were also validated by quantitative RT-PCR analysis. The data obtained in this study provide an excellent resource for unraveling the molecular mechanisms of browning processes during *in vitro* tissue culture, and lay a foundation for future studies to inhibit and eliminate browning damage.

## Introduction

Tissue culture *in vitro* has been an important technique for plant reproduction and germplasm conservation for half a century, and is a valuable tool for research as well as commercial germchit production (Morel, 1960; Thorpe, 1978; Kutchan, 1998; Loyola-Vargas and Vázquez-Flota, 2006). However, the plant browning response that frequently occurs is one of the most recalcitrant problems encountered. Browning has deleterious effects on plants and may lead to decreased regenerative ability, poor growth and even death of explants or cultures (Tang and Newton, 2004; He et al., 2009; Xu et al., 2011). This problem restricts the application of tissue culture technology in many species (Ahmad et al., 2013). Although browning damage may sometimes be alleviated by media supplements (active carbon, ascorbic acid, polyvinyl pyrrolidone, etc.), the results are not always satisfactory especially in some species that easily brown (Lee and Whitaker, 1995; Ahmad et al., 2013). A radical solution to this problem therefore requires a comprehensive understanding of the browning response.

Generally, plants with higher secondary metabolite contents are more prone to browning and more difficult to culture *in vitro* (Miller and Murashige, 1976; Dalal et al., 1992). For instance, severe browning occurs in *Medinilla* because of its abundant phenols and tannins, making it one of the most difficult genera for tissue culture (Casteele et al., 1981; Wang et al., 2015). Several studies have suggested that browning and the subsequent death of explants depends on the enzymatic oxidation of phenolic compounds, since the resulting quinones are highly reactive and toxic to plant tissue (Tomás-Barberán et al., 1997; Saltveit, 2000; Titov et al., 2006). The redox reaction is thought to be facilitated by the disorganization of cellular components, which destroys the localized distribution of polyphenol and polyphenol oxidase (Lee and Whitaker, 1995). Increasingly, researchers have proposed that browning might be a plant physiological response directly induced by environmental stress, such as temperature (Hoque and Mansfield, 2004), osmotic pressure (Dörnenburg and Knorr, 1997), nutrient stress (Rajendran et al., 1992), and hormones (Mohamed and Jayabalan, 1996; Moon et al., 2015). With the progress of molecular biology, the molecular mechanism of plant browning response has also been explored. Hirimburegama and Gamage (1997) found that *Musa* cultivars with genome ‘B’ had a more serious browning response and lower multiplication than other genotypes, while a map-based cloning strategy showed that the *Ic1* gene (Induced callus 1) controls callus browning of rice (Li et al., 2007). A proteomic study showed that mitochondrial ATPase and peroxiredoxin were only expressed in browning *Phalaenopsis* leaf explants cultured *in vitro* (Chen et al., 2012). However, there have so far only been a few studies of the genes responsible for browning and the mechanism of the browning response is not sufficiently clear. In addition, most previous research on plant browning has used explants subjected to mechanical wounding and disinfectant damage, which may themselves interfere with the normal browning response (Debergh and Maene, 1981; Ko et al., 2009). Therefore, the establishment of an aseptic system to avoid other interfering factors seems necessary if the molecular mechanism of browning is to be properly investigated.

Next generation sequencing has become a rapid and cost-effective means to analyze the genome and transcriptome of non-model species (Imelfort and Edwards, 2009; Wang et al., 2009). The present study used intact aseptic plantlets of *Medinilla formosana* Hayata previously obtained by ovary culture which have a severe browning response when cut (Wang et al., 2015). The changes in shoot morphology and histology after cutting were first observed and then *de novo* transcriptome and subsequent digital gene expression (DGE) profiling at different times after cutting were used to explore the genes and gene networks that play roles in regulating the browning response and plant growth. This study sheds fresh light on plant browning on a genome-wide scale and will facilitate future studies of its molecular modulation and effective inhibition.

## Materials and Methods

### Plant Materials and Cutting Treatment

Aseptic plantlets of *M. formosana* Hayata obtained through ovary culture were used as source materials (Wang et al., 2015). Small shoots were separately excised and immediately placed on half-strength (1/2) MS basal medium (Murashige and Skoog, 1962) supplemented with 3% sucrose and 0.8% agar (pH = 5.5) at 25 ± 2°C under a 16 h photoperiod, 40 μmol/m^2^⋅s fluorescent light. Subsequent morphological changes to the shoots were observed and photographed.

Plant materials of *M. formosana* for further molecular study were multiplied from a single plantlet to ensure genotypic consistency. The propagating medium was 1/2 MS basal medium supplemented with 3% sucrose, 0.8% agar (pH = 5.5), 4.44 μM 6-benzyladenine, 0.11 μM α-naphthaleneacetic acid, 0.5 g/l AC and 0.5 mM β-mercaptoethanol (as the only known effective inhibitor for the browning of *M. formosana*; Wang et al., 2015).

### Transmission Electron Microscopy (TEM)

Three replicate specimens (2 mm × 6 mm) were sampled from the middle part of the third leaf from the shoot tip 0, 2, 6, 15, 22, and 54 days after cutting (DAC) and then fixed with 2.5% glutaraldehyde for 1 h. After rinsing with buffer three times (15 min each), the samples were fixed in 1% osmium tetroxide for 2 h and again rinsed with buffer three times. Both fixing agents were prepared in 100 mM phosphate buffer (pH = 7.0). After dehydration in an alcohol series (50, 70, 80, 90, 95, and 100% for 15 min each) and acetone (20 min twice) the specimens were infiltrated with 30% (in acetone), 70% and pure Spurr resin. After polymerization for 24 h at 70°C, semi-thin sections (1 μm) were cut transversely and observed under an optical microscope (Olympus SP 350). Ultra-thin leaf sections (80 nm) for transmission electron microscopy were cut transversely using a UC6 microtome (LEICA EM). After staining with uranyl acetate and lead citrate for 20 min each, sections were examined with an H-7650 transmission electron microscope (Hitachi, Japan) at 80 kV. Photographs of interest were taken by a Gatan 830 CCD camera (Gatan, USA).

### Sample Collection and RNA Isolation

Shoot samples (about 0.1 g containing leaf and stem) were collected 0 hour after cutting (HAC), 4 HAC, 2 DAC, and 8 DAC, frozen in liquid nitrogen and stored at -80°C. Total RNA was isolated using the improved CTAB method. RNA degradation and contamination was monitored on 1% agarose gels, RNA purity was checked using the Nano-Photometer^®^ spectrophotometer (IMPLEN, Westlake Village, CA, USA), RNA concentration was measured using Qubit^®^ RNA Assay Kit in Qubit^®^2.0 Fluorometer (Life Technologies, Camarillo, CA, USA) and RNA integrity was assessed using the RNA Nano 6000 Assay Kit of the Bioanalyzer 2100 system (Agilent Technologies, Santa Clara, CA, USA).

### Library Preparation and Transcriptome Sequencing

A total of 3 μg RNA mixed from the four time samples was used as input material for the RNA sample preparations. Sequencing libraries were generated using the NEBNext^®^ Ultra^TM^ RNA Library Prep Kit for Illumina^®^ (NEB, USA) following the manufacturer’s recommendations and index codes were added to attribute sequences to each sample. Briefly, mRNA was purified from total RNA using poly-T oligo-attached magnetic beads. Fragmentation was carried out using divalent cations under elevated temperature in NEBNext First Strand Synthesis Reaction Buffer (5X). First strand cDNA was synthesized using random hexamer primers and M-MuLV Reverse Transcriptase (RNase H^-^). Second strand cDNA synthesis was subsequently performed using DNA Polymerase I and RNase H. Remaining overhangs were converted into blunt ends by exonuclease/polymerase. After adenylation of the 3′ ends of DNA fragments, NEBNext Adaptors with hairpin loop structures were ligated to prepare for hybridization. To preferentially select cDNA fragments of 150–200 bp, the library fragments were purified using the AMPure XP system (Beckman Coulter, Beverly, MA, USA). Then 3 μl USER Enzyme (NEB, USA) was used with size-selected, adaptor-ligated cDNA at 37°C for 15 min followed by 5 min at 95°C before PCR. Then PCR was performed with Phusion High-Fidelity DNA polymerase, Universal PCR primers and Index (X) Primer. Finally, PCR products were purified (AMPure XP system) and library quality was assessed on the Agilent Bioanalyzer 2100 system.

The clustering of the index-coded samples was performed on a cBot Cluster Generation System using TruSeq PE Cluster Kit v3-cBot-HS (Illumina) according to the manufacturer’s instructions. After cluster generation, the library preparations were sequenced on an Illumina Hiseq 2000 platform and paired-end reads were generated.

### Quality Control, Transcriptome Assembly, and Functional Annotation

Raw reads in fastq format were first processed through in-house perl scripts to generate clean reads by removing low quality reads and those containing adapters or poly-N. Simultaneously, Q20, Q30, GC-content, and sequence duplication levels of the clean data were calculated. All the downstream analyses were based on high quality clean data. Transcriptome assembly was accomplished based on the left.fq and right.fq using Trinity (Grabherr et al., 2011) with min_kmer_cov set to 2 by default and all other parameters set to default.

Gene function was annotated based on the following databases: Nr (NCBI non-redundant protein sequences), Nt (NCBI non-redundant nucleotide sequences), Pfam (Protein family), KOG/COG (Clusters of Orthologous Groups of proteins), Swiss-Prot (A manually annotated and reviewed protein sequence database), KO (KEGG Ortholog database), GO (Gene Ontology).

### Activity Assessment of Known Enzymes Related to Browning

The activity changes of enzymes related to the phenolic redox reaction after cutting were first measured to determine the appropriate sampling stage for DGE profiling. For phenylalanine ammonia-lyase (PAL) and polyphenol oxidase (PPO), four replicate samples of 0.2 g leaf tissue of *M. formosana* were taken 0 h, 4 h, 12 h, 1 days, 2 days, 4 days, and 8 days after cutting and stored at -20°C. Enzyme activity was determined on an ultraviolet spectrophotometer using the Comin PAL and PPO Kit (Cominbio, China) according to the manufacturer’s instructions. Peroxidase (POD) activity was similarly determined but using 0.1 g leaf tissue and the Comin POD Kit (Cominbio, China).

### Comparative DGE Profiling

On the basis of the enzyme activities determined as described above, RNA samples were, respectively, extracted from the replicated shoots at 0 HAC (cut_0h1 and cut_0h2), 4 HAC and 4 DAC (cut_4h1 and cut_4h2, cut_4d1 and cut_4d2). Totally six DGE libraries were prepared and sequenced as described for the transcriptome sequencing above. In addition to the normal quality control, the correlation between the counts from parallel libraries was assessed using the Pearson correlation coefficient calculated in Microsoft Excel. Clean data were mapped back onto our assembled transcriptome reference database of *M. formosana*, and the read-count for each gene was obtained from the mapping results using RSEM software. Gene expression was normalized to RPKM (reads per kb per million mapped reads), to eliminate the influence of different gene lengths and sequence discrepancies on the calculation of gene expression (Mortazavi et al., 2008).

### Identification and Annotation of Differentially Expressed Genes

Differentially expressed genes were identified using the DESeq R package (1.10.1). This provides statistical routines for determining DGEs using a model based on the negative binomial distribution. The resulting *P*-values were adjusted using the Benjamini and Hochberg’s approach for controlling the false discovery rate (FDR). In our study, genes meeting the stringent criteria of *P*-value ≤ 0.005 and log_2_ (foldchange) ≥ 1 were assigned as DEGs. Gene expression was further normalized to FPKM (fragments per kb of exon per million mapped reads), and log10(FPKM+1) values were used for hierarchical clustering of common DEGs by pheatmap packages^1^.

Differentially expressed genes were annotated as described by Zhao et al. (2013). GO enrichment analysis of putative genes was implemented by the GOseq R package, in which gene length bias was corrected (Götz et al., 2008; Young et al., 2010). KEGG is a database resource for understanding high level functions and utilities of the biological system, such as the cell, the organism, and the ecosystem, from molecular-level information, especially large-scale molecular datasets generated by genome sequencing and other high-throughput experimental technologies^2^ (Kanehisa et al., 2008). We used KOBAS software to test the statistical enrichment of putative genes in KEGG pathways.

### qRT-PCR Analysis

RNAs for qRT-PCR analysis were extracted from three replicate samples each of shoots at 0 DAC, 4 HAC, 1 DAC, and 4 DAC as described above. Primer sequences were designed by Primer 5.0 and synthesized by Generay Biotech (Generay, PRC) based on the mRNA sequences obtained from transcriptome sequencing. Reverse transcription reactions were performed in a GeneAmp^®^ PCR System 9700 (Applied Biosystems, USA) for 15 min at 37°C, followed by heat inactivation of RT for 5 s at 85°C. The 10 μl RT reaction mix was then diluted × 10 in nuclease-free water and held at -20°C. Real-time PCR was performed using a LightCycler^®^ 480 II Real-time PCR Instrument (Roche, Swiss) with 10 μl PCR reaction mixture that included 1 μl of cDNA, 5 μl of 2 × LightCycler^®^ 480 SYBR Green I Master (Roche, Swiss), 3.6 μl of nuclease-free water, and 0.2 μl of each primer. Reactions were incubated in a 384-well optical plate (Roche, Swiss) at 95°C for 10 min, followed by 40 cycles of 95°C for 10 s, 60°C for 30 s. Each sample was run in triplicate. At the end of the PCR cycles, melting curve analysis was performed to validate the specific generation of the expected PCR product. The expression levels of mRNAs were normalized to 18sRNA and were calculated using the 2^-ΔΔCt^ method (Livak and Schmittgen, 2001).

## Results

### Effects of Browning on *M. formosana* after Cutting

**FIGURE 1:**
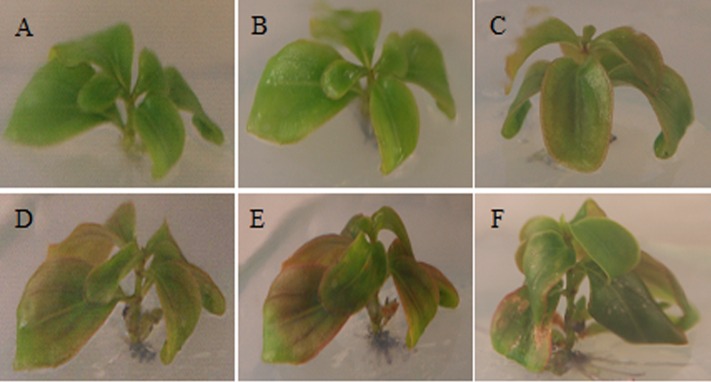
**Morphological changes in *Medinilla formosana* shoots after cutting. (A)** Shoot at 0 day after cutting (DAC). **(B)** Shoot at 2 DAC, The leaf color began to turn brownish red from the margin. **(C,D)** Shoot at 4 and 6 DAC. The color of leaves gradually deepened and spread to the leaf center later. **(E)** Shoot at 15 DAC. The developed leaves in the middle section were obviously copper-red. **(F)** Shoot at 22 DAC.

Totally 34 plantlets of *M. formosana* cultured *in vitro* were monitored to characterize the browning process in detail. As shown in **Figure 1**, visible browning at the cutting site occurred at 2 DAC in all plantlets. The leaf began to turn red from the margin, gradually spreading to the center. The most obvious copper-red color appeared in the middle of developed leaves at about 15 DAC and partial necrosis appeared at about 22 DAC. After this, new green leaves sprouted and the whole plantlets seemed to start to recover.

**FIGURE 2:**
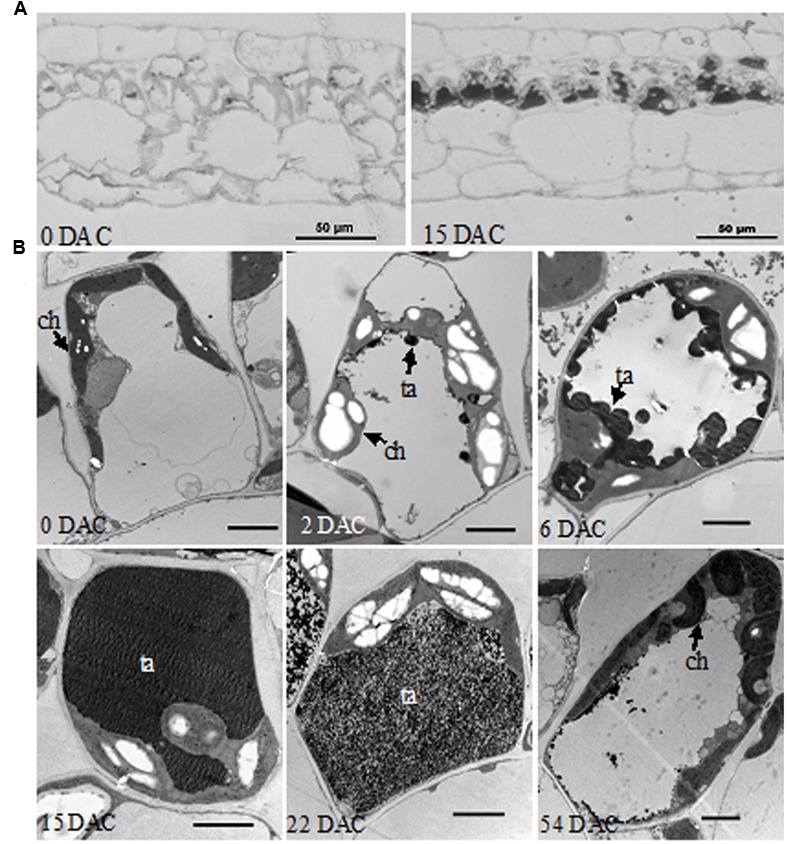
**Histological observations of *M. formosana* leaves after cutting. (A)** Semi-thin section of leaves at 0 and 15 DAC under the light microscope, bar = 50 μm; **(B)** Transmission Electron Microscopy (TEM) observations of palisade tissue cells. ch, chloroplast; ta, tannins; bar = 2 μm.

In semi-thin resin sections of leaves, an abundant black substance accumulated in palisade tissue cells at 15 DAC, while little was found in the control leaves at 0 DAC (**Figure 2A**). In further TEM, clumps of osmiophilic condensed tannins were found at the edge of vacuole at 2 DAC (Casteele et al., 1981; Laukkanen et al., 1999); while excess starch grains appeared in the chloroplasts and caused them to swell significantly. Subsequently, condensed tannins continued to increase toward the center of the vacuole and the cells gradually became spherical. At 15 DAC, the vacuole was completely filled with dark tannins, and most organelles seemed to be squeezed to the border of cytoplasm. In contrast, palisade cells at 0 DAC were almost rectangular in section, and their organelles were well-organized (**Figure 2B**). However, condensed tannins began to disaggregate at 22 DAC and almost vanished at 54 DAC. The excess starch grains in chloroplasts also disappeared and the whole cells, and their organelles, recovered to a normal appearance.

### *De novo* Transcriptome Sequencing, Assembly, and Annotation

**FIGURE 3:**
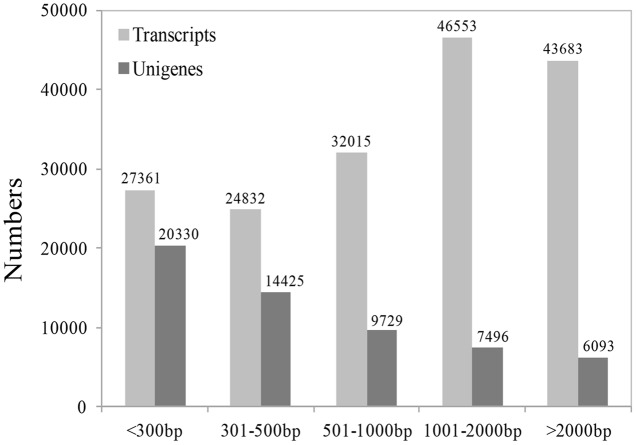
**The length distribution of unigenes and transcripts**.

Because there are no EST data and little genomic sequence from *Medinilla* species (Clausing and Renner, 2001; Zeng et al., 2016), we first sequenced the transcriptome at four stages after cutting using the Illumina sequencing platform. An overview of the sequencing and assembly is summarized in **Table 1**; **Figure 3**. After cleaning and quality checks, about 7.5 G clean reads were obtained and spliced into transcripts by Trinity. A total of 174,444 transcripts were obtained, with average length of 1,403 bp, while 58,073 unigenes were identified and their average length was 810 bp (**Table 1**). An analysis of the lengths of transcripts and unigenes is presented in **Figure 3**. There were 90,236 transcripts (51.73% of all transcripts) with length over 1000 bp and 20,330 unigenes (45.09% of all unigenes) with length under 301.

**Table 1:** Overview of the *Medinilla formosana* transcriptome.

Total number of raw reads	80367590
Total number of clean reads	75,680,870
GC percentage	49.34%
Total Nucleotides	244,830,224
Total number of transcripts	174,444
Mean length of transcripts	1,403
Min length of transcripts	201
Max length of transcripts	16,751
Total number of unigenes	58,073
Mean length of unigenes	810

Multiple databases were interrogated to accurately annotate the unigenes, including the NCBI database Non-redundant protein sequences (Nr), the manually annotated and curated protein sequence database (Swiss-Prot), NCBI nucleotide sequences (Nt), Protein Family (Pfam), euKaryotic Ortholog Groups (KOG), GO, and Kyoto Encyclopedia of Genes and Genomes Ortholog database (KO). As shown in **Table 2**; **Supplementary Table S1**, 26,022 unigenes (44. 81% of all unigenes) showed significant similarity to known proteins in the NR database and a total of 28,433 proteins were annotated. However, only 2580 unigenes (4.44% of all unigenes) could be annotated from these seven databases.

**Table 2:** Unigene annotation of *M. formosana* transcriptome.

Annotation database	Number of unigenes	Percentage (%)
Annotated in NR	26,022	44.81
Annotated in NT	8,101	13.94
Annotated in KO	7,918	13.63
Annotated in Swiss-Prot	18,820	32.41
Annotated in PFAM	18,134	31.22
Annotated in GO	20,674	35.60
Annotated in KOG	9,394	16.17
Annotated in all databases	2,580	4.44
Annotated in at least one database	28,433	48.96

*NR, NCBI non-redundant protein sequences; NT, NCBI nucleotide sequences; KO, KEGG Ortholog; Swiss-Prot, A manually annotated and reviewed protein sequence database; PFAM. Protein family; GO, Gene Ontology; KOG, euKaryotic Ortholog Groups.*

### DGE Profiling at Different Stages after Cutting

**FIGURE 4:**
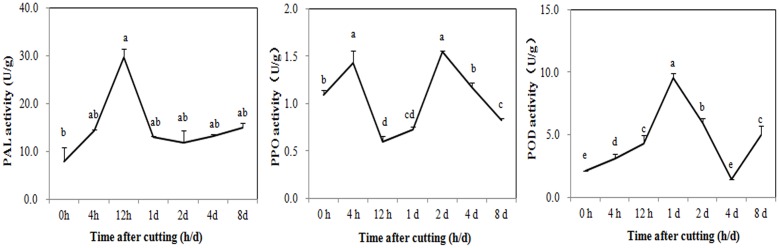
**Activity changes of PAL, PPO, and POD in *M. formosana* after cutting treatment.** Mean activity of phenylalanine ammonia-lyase (PAL), polyphenol oxidase (PPO), and Peroxidase (POD) are shown with standard errors bars from four replicates. Points labeled with same letter are not significantly different from one another according to a LSD test at *P* < 0.05.

Digital gene expression profiling was then used to explore the expression changes of genes involved in browning. Changes in the activity of enzymes related to the phenolic redox reaction after cutting were first measured to choose the appropriate sampling stages for DGE profiling. As shown in **Figure 4**, PAL was rapidly activated to 29.72 U/g at 12 HAC and declined at 1 DAC, while the activity of PPO has two peaks at 4 HAC and 2 DAC. POD activity peaked at 1 DAC (9.51 U/g) and then dramatically decreased at 2 and 4 DAC. To efficiently screen the key genes modulating the browning response, RNA samples were therefore extracted from the shoots at 0 HAC, 4 HAC, and 4 DAC (cut_0h, cut_4h and cut_4d) for DGE profiling.

**FIGURE 5:**
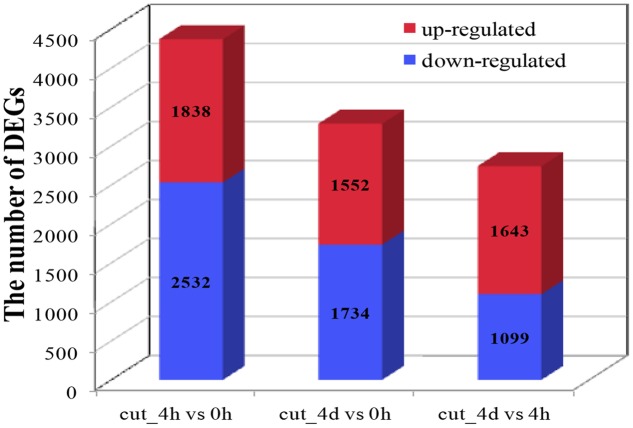
**Statistics of Differentially expressed gene (DEG) numbers at different culture stages.** The numbers of up-regulated and down-regulated genes in comparisons between two stages after cutting are displayed (cut_4h vs cut_0h, cut_4d vs cut_0h, and cut_4d vs cut_4h).

About seven million raw reads per library were generated, with clean reads of the six libraries ranging from 6.0 to 7.1 million. About 92% of the tag sequences could be mapped to our *de novo* transcriptome database, which showed the high reliability of the reference transcriptome data (**Table 3**). Additionally, Pearson correlation analysis for two biological replicates of the cut_0h, cut_4h and cut_4d samples confirmed the reproducibility of library sequencing: the indexes of Pearson correlation (R^2^) for the parallel libraries were 0.9, 0.879, and 0.892, respectively (**Supplementary Figure S1**). The DGE profiling identified 3,370 genes between cut_4h and cut_0h, of which 1,838 genes were significantly up-regulated and 2,532 genes were down-regulated (**Figure 5**). Totally 3286 genes were differentially expressed between cut_4d and cut_0h, with 1,552 genes up-regulated and 1,734 genes down-regulated; 1,779 genes were differentially expressed between cut_4d and cut_4h, with 1,643 genes up-regulated and 1,099 genes down-regulated (**Figure 5**).

**Table 3:** Overview of digital gene expression (DGE) analysis after cutting.

Sample	Raw reads	Total reads	Mapped reads	Percentage (%)
cut_0h1	6,825,071	6,642,427	6,130,866	92.30
cut_0h2	6,371,775	6,099,595	5,625,771	92.23
cut_4h1	6,416,088	6,177,170	5,686,169	92.05
cut_4h2	7,429,294	7,126,093	6,559,014	92.04
cut_4d1	7,326,096	7,040,432	6,502,282	92.36
cut_4d2	7,164,566	6,970,542	6,421,580	92.12

### Expression Pattern Analysis of DEGs

**FIGURE 6:**
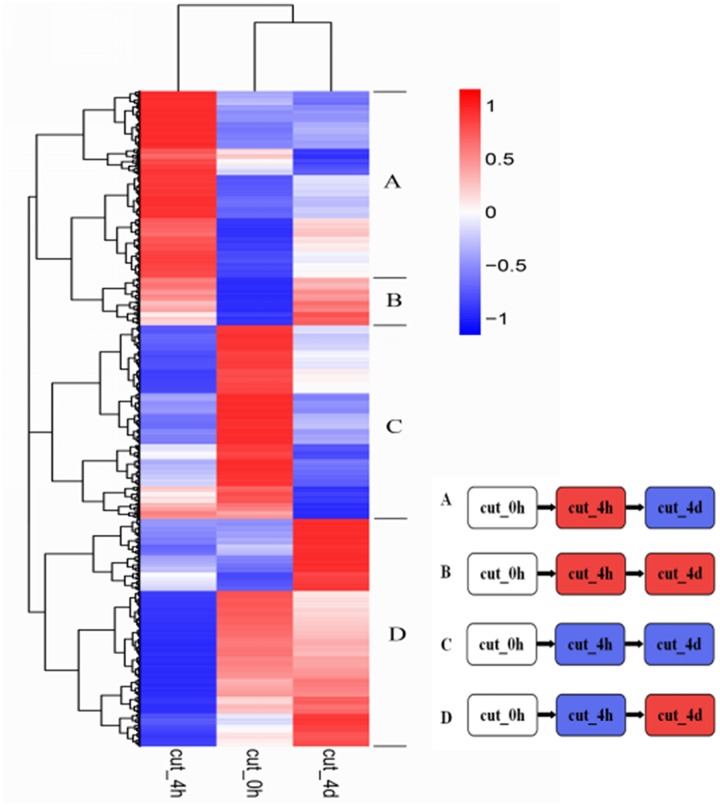
**Hierarchical cluster analyses of 6,431 DEGs in *M. formosana* after cutting.** Each column represents a sampling stage after cutting (e.g., cut_0h), each row represents a gene expression ratio expressed as log_10_ (relative gene expression values). Red represents up-regulation and blue represents down-regulation of expression compared with the adjacent stage. The expression patterns of all DEGs could be allocated to one of four sub-clusters labeled A–D.

Hierarchical clustering of the 6,431 DEGs common to the three stages was performed to examine the similarity and diversity of expression profiles (**Figure 6**). This provides a global view of the gene expression pattern of DEGs during the early stage of browning. To further study the genes functioning during browning of *M. formosana*, all DEGs were roughly categorized into four sub-clusters according to their expression pattern at the three stages (**Figure 6**) and most DEGs with specific and typical expression patterns are identified in **Supplementary Table S2**. Pattern A contains 931 DEGs with significant up-regulation at 4 HAC [log_2_ (cut_4h : cut_0h) > 1] and down-regulation at 4 DAC [log_2_ (cut_4d : cut_4h) < 1], and Pattern D consists of 1179 DEGs with down-regulation at 4 HAC and up-regulation at 4 DAC. Patterns B and C were genes, respectively, up- and down-modulated throughout all three stages, and comprised 116 and 122 genes. All these genes were significantly regulated and may contribute to the early modulation of browning.

**FIGURE 7:**
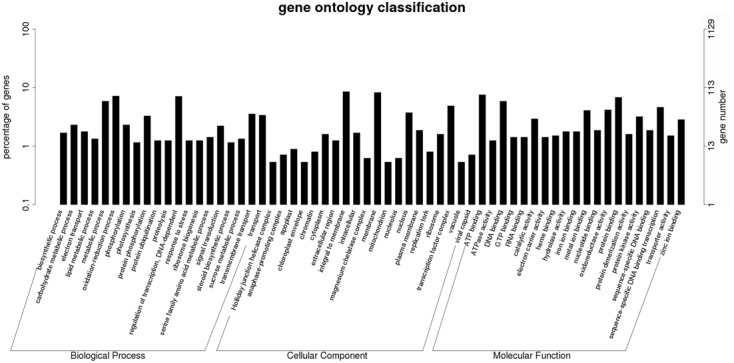
**Histogram of Gene Ontology classifications of DEGs in Sub-cluster A.** The results are summarized in three main categories: biological process, cellular component, and molecular function. The left *y*-axis indicates the percentage of a specific category of genes in total genes, and the right *y*-axis indicates the number of genes in a category.

**FIGURE 8:**
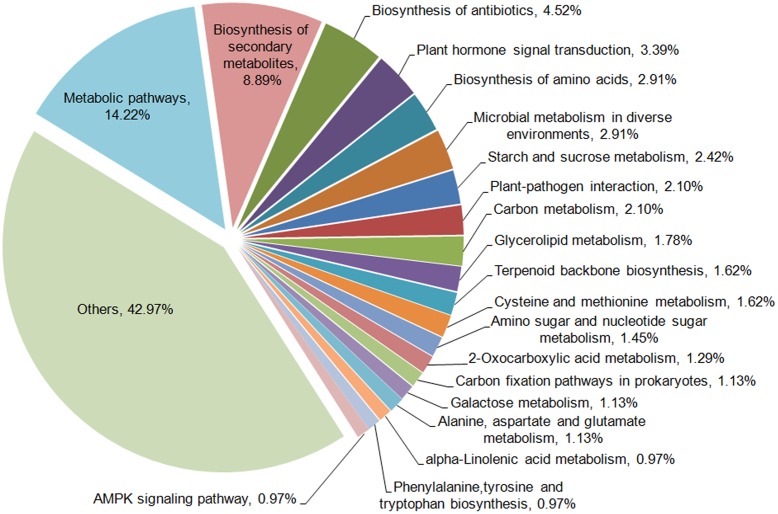
**KEGG enrichment classifications of DEGs in Sub-cluster A**.

To further examine the DEGs showing different patterns during browning, genes of pattern A were first functionally annotated, as these are probably involved in the metabolic or signal transduction pathways in the early steps of browning. GO is a classification system to describe the properties of the organism genes and their products, including biological process, cellular component, and molecular function. A total of 694 genes were annotated with the three GO ontologies and the top 20 terms are shown in **Figure 7**. The subcategories of “transcription regulation,” “metabolic process,” “oxidation-reduction process,” and “transmembrane transport” were dominant in the biological process category. In the cellular component category, “integral to membrane” and “membrane” were highly represented. In the molecular function category, “ATP, DNA, and protein binding” and “oxidoreductase activity” were dominant subcategories. Furthermore, KEGG enrichment classification (hypergeometric test) was used to identify significantly enriched pathways of DEGs between the different stages of browning using a FDR ≤ 0.05. A total of 172 DEGs were annotated with 154 terms and the top 20 KEGG pathways are shown in **Figure 8**. The most highly represented was ‘metabolic pathways’ (ko01100) and ‘biosynthesis of secondary metabolites’ (ko01110), which accounted for 14.22 and 8.89%, respectively, followed by ‘biosynthesis of antibiotics’ (ko01130) and ‘plant hormone signal transduction’ (ko04075) with, respectively, 4.54 and 3.39%. Additionally, lipid (linolenic acid and glycerolipid) and amino acid (alanine, aspartate, glutamate, cysteine, and methionine) metabolism were also involved.

The top10 DEGs of Patterns B, C, and D were also listed according to their absolute values of fold change and annotated. As shown in **Table 4**, most DEGs of Pattern B were found to be related to the biosynthesis and oxidation process of phenolics (comp40461_c0, comp57388_c0, comp36974_c0, comp41826_c1, and comp63629_c0), transcription regulation (comp58041_c0), and sugar metabolic process (comp56210_c0, comp56210_c0). Genes of Pattern C were mainly involved in nutrient transport (comp58069_c1, comp60016_c0, comp51640_c0, and comp41083_c0), carbohydrate metabolic process (comp66737_c0, comp64795_c0, comp54196_c1, and comp66048_c0), and negative regulation of transcription (comp57423_c0). The majority of genes of Pattern D participate in photosynthesis (comp56595_c0, comp38163_c0, and comp53042_c0), energy metabolism (comp64552_c1), and response to stress (comp46179_c0 and comp53783_c0). The sequence data of our DGE profiling has been deposited into the NCBI Sequence Read Archive (SRA) database with accession number SRA495968.

**Table 4:** Top 10 DEGs showing prominent changes after cutting.

Gene ID	FPKM			Gene description
	0 h	4 h	4 d	
**Sub-cluster B**				
comp58041_c0	0.00	0.07	4.52	Zinc finger protein, putative [*Ricinus communis*]
comp40461_c0	0.00	0.13	3.44	PREDICTED: laccase-12-like [*Vitis vinifera*]
comp54854_c0	0.00	0.36	10.86	Lipid binding protein, putative [*Ricinus communis*]
comp56210_c0	0.00	0.3	5.29	PREDICTED: putative invertase inhibitor [*Vitis vinifera*]
comp41826_c1	0.00	0.45	12.85	Hypothetical protein POPTRDRAFT_554508 [*Populus trichocarpa*]
comp34094_c0	0.00	1.65	7.40	Sugar transporter, putative [*Ricinus communis*]
comp63629_c0	0.27	10.87	37.93	2-alkenal reductase [*Arabidopsis thaliana*]
comp48947_c0	0.51	16.75	51.93	CINNAMYL alcohol dehydrogenase-like protein [*Populus trichocarpa*]
comp57388_c0	0.00	0.56	25.49	Conserved hypothetical protein [*Ricinus communis*]
comp36974_c0	0.20	3.36	33.02	Flavonoid 3′-hydroxylase, partial [*Morella rubra*]
**Sub-cluster C**				
comp58069_c1	4.84	0.49	0.19	PREDICTED: metal transporter Nramp5 [*Vitis vinifera*]
comp66737_c0	180.98	35.50	6.93	Beta-galactosidase, putative [*Ricinus communis*]
comp64795_c0	42.18	10.23	2.52	Hypothetical protein VITISV_039434 [*Vitis vinifera*]
comp54196_c1	2.74	0.71	0.21	Hydrolase, putative [*Ricinus communis*]
comp41083_c0	16.36	0.37	0.00	PREDICTED: monothiol glutaredoxin-S2-like [*Glycine max*]
comp60016_c0	2.49	0.95	0.31	Sugar transporter, putative [*Ricinus communis]*
comp66048_c0	55.67	22.17	7.26	Beta-amylase [*Castanea crenata*]
comp59756_c0	177.30	75.79	12.07	Fatty acid hydroperoxide lyase [*Psidium guajava*]
comp51640_c0	4.08	1.81	0.71	Pentatricopeptide repeat-containing protein At5g55740 [*Vitis vinifera*]
comp57423_c0	4.41	1.82	0.76	PREDICTED: protein SRG1 [*Vitis vinifera*]
**Sub-cluster D**				
comp60636_c0	8.73	0.07	4.39	Predicted protein [*Populus trichocarpa*]
comp64552_c1	43.20	0.95	5.46	Fructose-bisphosphate aldolase, putative [*Ricinus communis*]
comp46807_c0	1.67	0.06	2.09	Cytochrome P450, putative *[Ricinus communis*]
comp59471_c0	2.35	0.08	4.34	Predicted protein [*Populus trichocarpa*]
comp47029_c0	12.88	0.42	2.60	Expansin18 precursor [*Solanum lycopersicum*]
comp56595_c0	14.68	0.83	19.61	Copper binding protein 3 [*Gossypium hirsutum*]
comp46179_c0	757.45	28.13	540.00	Predicted protein [*Populus trichocarpa*]
comp53783_c0	1.82	0.1	0.81	Hypothetical protein MTR_5g047050 [*Medicago truncatula*]
comp38163_c0	81.15	4.43	10.49	Chlorophyll a/b binding protein [*Solanum tuberosum*]
comp53042_c0	19.87	0.92	8.60	Light-harvesting complex II protein Lhcb6 [*Populus trichocarpa*]

*The genes were selected according to the absolute value of foldchange and stored by p-value < 0.05. FPKM means expected number of fragments per kilobase of transcript sequence per millions base pairs sequenced.*

### Validation of DGE Data by qRT-PCR

**FIGURE 9:**
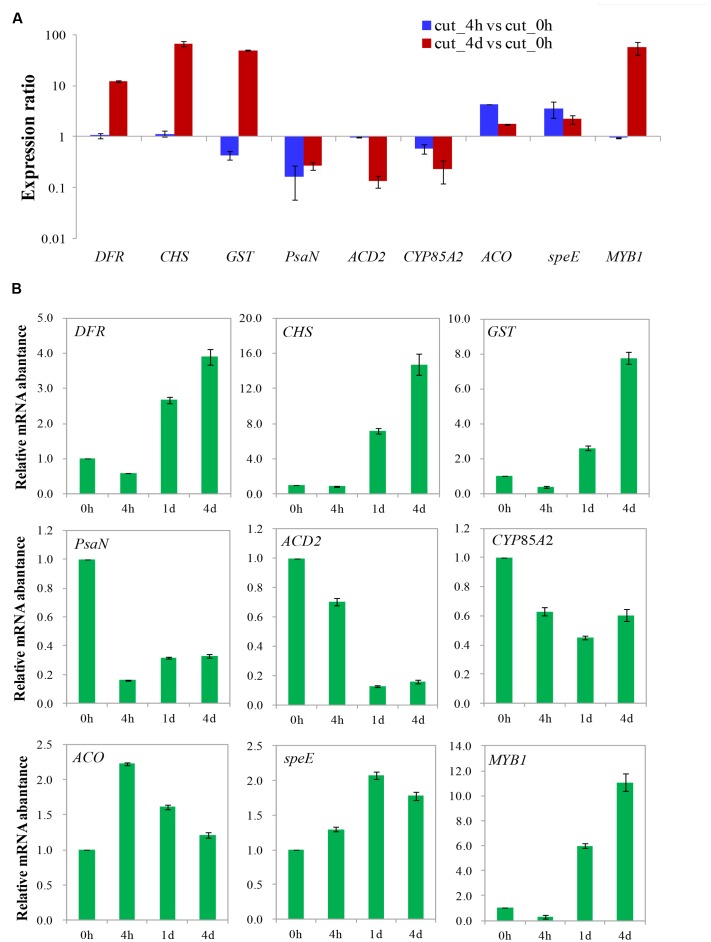
**Validation of differential gene expression of after cutting.** Gene expression ratios obtained from RNA-seq **(A)** and relative mRNA abundance from qRT-PCR **(B)** data of different times after cutting.

A total of nine genes with various expression patterns were selected from the four DEGs clusters to validate the DGE data by qRT-PCR. The actin gene 18sRNA from transcriptome data of *M. formosana* was used as an internal control, and the primers employed in the qRT-PCR are listed in **Supplementary Table S3**. The relative quantitative method (2^-ΔΔCt^) was used to calculate the fold change of the target genes. As shown in **Figure 9**, the qRT-PCR data for these genes were generally consistent with the DGE results. Moreover, linear regression [(DGE value) = a(qRT-PCR value)+b] analysis showed a correlation coefficient of 0.7619, indicating that a positive correlation between the DGE data and qRT-PCR data (**Supplementary Figure S2**). Although the observed expression differed slightly between qRT-PCR and DGE data, this may reflect differences in the sensitivity and specificity of qRT-PCR and high-throughput sequencing technology.

## Discussion

### The Browning of *M. formosana* Is Not Lethal but Self-Adaptive

Although visible and anatomical studies of brown tissues have been reported previously, the dynamics of browning on plants has been less clear (Tang and Newton, 2004; Xu et al., 2011). In this study, successive morphological and intracellular observation of *M. formosana* after cutting supplied a more comprehensive characterization of the browning response (**Figures 1** and **2**). Generally, it is suggested that the browning at the local cutting site might affect plant absorption of water, mineral and nutriment from medium during tissue culture due to the blocking of vascular bundles (Xu et al., 2015a). Besides, anthocyanin and condense tannins were found accumulated in the leaves of excised shoots, which are considered common responses of plant enhancing resistance to biotic and abiotic stress (Clausen et al., 1992; Rajendran et al., 1992; Arnold et al., 2008). In addition, the swelling of chloroplasts and the excess of starch grains in their leaves were also observed. Similar phenomena have been reported in plant callus subjected to adverse environmental conditions, such as the temperature, osmotic, and nutrient stress (Rajendran et al., 1992; Laukkanen et al., 1999; Hong et al., 2014). Importantly, the browning response faded after a certain period and all plantlets gradually recovered, with the depolymerization of condensed tannins and recovery of the organelles. It can be inferred that the browning was not lethal and may be a complicated self-adaptive process to the wound stress. The death of browning explants reported in previous studies on *Medinilla* may therefore be caused by multiple factors, such as the damage caused by disinfection and inappropriate culture environment (Reinert and Bajaj, 1977; Casteele et al., 1981).

For a relatively long time, browning response has usually been attributed to the accumulation of phenolic compounds and their enzymatic oxidation (Tomás-Barberán et al., 1997; Saltveit, 2000; Titov et al., 2006). However, there are many studies suggesting that browning may be a physiological response directly induced by abiotic stress rather than enzymatic reaction of phenolics (Ke and Saltveit, 1989; Saltveit, 2000; Moon et al., 2015). The osmotic stress caused by chitosan can induce browning of potato suspension cells, with an increase in phenolic content and in the activity of PAL and PPO (Dörnenburg and Knorr, 1997). Wu and Lin (2002) reported that ultrasound-induced stress gave rise to browning in *Panax ginseng* cells, while drought stress induced by polyethylene glycol induced browning in sugarcane and beet (Arencibia et al., 2012; Sen and Alikamanoglu, 2013). In our study, all wounded plantlets of *M. formosana* displayed browning on their leaves whether the cutting was done to stem, leaf, or root, while the intact plantlets cultured on the same medium had no browning (data not shown). It therefore seems likely that the mechanical wound initially elicits browning as a kind of abiotic stress, although further molecular evidence is needed.

### Browning-Related Genes with Different Expression Patterns after Cutting

*De novo* transcriptome sequencing of *M. formosana* and subsequent DGE profiling after cutting were performed here using the Illumina HiSeq 2000 platform for the first time. This provides an opportunity to study the molecular modulation of *M. formosana* during the browning that occurs after cutting. DEGs with various expression patterns at different stages were identified and roughly categorized into four sub-clusters (**Figure 6**), which helps us methodically study the genes and gene networks that play roles in regulating browning.

The DEGs with most up-regulation throughout all stages are mostly those involved in the synthesis of phenolic compounds and related oxidoreductases. It seems that the accumulation of phenolic compounds and their enzymatic oxidation occurred during browning exactly as reported previously with other plant species (Veltman et al., 2003; Franck et al., 2007; Cascia et al., 2013). The consistently down-regulated DEGs were mainly involved in carbohydrate metabolism and nutrient transport, which implies that the growth of *M. formosana* was stunted after cutting to some degree. For example, β-amylase (comp66048_c0) has abundant activity for starch and amylopectin degrading and catalyses the liberation of β-maltose from the non-reducing end of α-1, 4-glucans (Baba and Kainuma, 1987; Bruns and Hecht-Buchholz, 1990; Scheidig et al., 2002; Parida et al., 2004). The suppression of β-amylase therefore presumably blocks starch metabolism in the leaves and results in the chloroplast-targeted starch-excess seen in *M. formosana* after cutting (**Figure 2**). Many of the DEGs in Pattern D are related to photosynthesis, energy metabolism and stress response. This suggests that the growth and metabolism of *M. formosana* was quickly blocked after cutting but then started to recover at 4 DAC. Meanwhile, the defense responses of plants were launched and resistance was elevated at 4 DAC. Cytochrome P450 is a large plant protein family playing an important role in the biosynthesis of secondary metabolites (steroid, glucosinolate, and phenylpropanoids, etc.) and plant defense to various stresses (Schuler and Werck-Reichhart, 2003). Its expression was significantly up-regulated after a short period of decreased modulation. A similar regulation pattern has been reported in browned lotus and *Phalaenopsis* tissue (Schuler and Werck-Reichhart, 2003; Jiang et al., 2012; Xu et al., 2015b). These results provide molecular evidence supporting the view that mechanical wounding induces browning as an abiotic stress and helps to identify the genes with changed expression profiles after cutting that are likely to play roles during browning in *M. formosana*.

### Identification of Putative Genes That May Play Signaling Roles in the Early Steps of Browning

Cutting of plants is usually inescapable during tissue culture. However, in our study there was not only browning at the cutting site but an obvious abnormal response in the leaves of *M. formosana*. How does leaf tissue perceive the local wound at an injured site on the stem and respond to it? A series of studies indicated that a mechanical wound can produce mobile plant signals, which rapidly respond locally to damage and also move to uninjured tissue giving plants the ability to activate and modulate defense pathways (Karban and Baldwin, 1997; Saltveit, 2000; Howe and Jander, 2008; Heil, 2009; Walling, 2009; Scranton et al., 2013). It is suggested that reactive oxygen species (ROS) including the superoxide anion are transiently produced soon after wounding and represent an initial step in local signaling at the wound site (Doke et al., 1991; Orozco-Cardenas and Ryan, 1999). Systemin, the first described mobile long-distance signal, also plays an essential role in the systemic wound-activated response, acting as a peptide defense elicitor produced in the cytoplasm of injured cells in young excised tomato plants and transmitted to others through the apoplast (Pearce et al., 1991; Ryan, 2000; Scheer and Ryan, 2002). In addition, oligogalacturonide, traumatin, proteinase inhibitors and hormonal molecules also functioned in signal transduction in wounded runner bean and tomato plants (Zimmerman and Coudron, 1979; Baydoun and Fry, 1985; Farmer and Ryan, 1992; Peña-Cortés et al., 1995; O’Donnell et al., 1996; Heil et al., 2012). However, the signal transduction involved in browning after cutting remains largely unstudied.

In order to understand the gene networks and signal transduction during browning in *M. formosana*, DEGs of Pattern A were selected for GO and KEGG enrichment analysis (**Figures 7** and **8**). It is well known that early changes in the activity of messengers regulated in plant resistance mechanisms are often mediated by phytohormones (Nürnberger and Scheel, 2001; Ha et al., 2012). The modulation roles of hormones in response to abiotic stresses including mechanical wounding has also been documented previously (Hare et al., 1997; Beaudoin et al., 2000; Griffiths et al., 2006; Tuteja, 2007; Hirano et al., 2008; Fariduddin et al., 2014), but the network of signal transduction during browning has been rarely reported until now. In the present study, auxin influx carrier protein (AUX1) on the plant plasmalemma seems to be the earliest element responding to wounding in *M. formosana* (**Supplementary Figure S3**) (Swarup et al., 2004; Yang et al., 2006). The brief expression of Aux/IAA, GH3, and small auxin-up RNAs (SAUR) were also detected, and these were considered the primary auxin-related genes that responded in previous studies (Hagen and Guilfoyle, 2002; Park et al., 2007, 2015; Singh et al., 2014). Modulation of other hormones were also found after cutting, indicating that browning of *M. formosana* is likely regulated by a complicated network of interconnecting signal transduction pathways of multiple hormones (**Supplementary Figure S3**). In previous studies, jasmonic acid (JA) and its derivatives [methyl jasmonate (MeJA) and jasmonoyl-isoleucine (JA-Ile)] were suggested to serve as systemic mobile signals that regulate proteinase inhibitor genes to respond to wounds caused by insect or pathogen attacks (Moura and Ryan, 2001; Heitz et al., 2012) (**Supplementary Figure S4**). Endogenous JA is rapidly induced by mechanical damage to leaves of *Arabidopsis*, maize, Lima beans, sesame, strawberry, tomato, etc. (Heil et al., 2012). Li et al. (2002) provided evidence that JA (or its derivatives) serves as a long-distance transmissible signal for wound signaling using two tomato mutants defective in the system in signaling pathway. In our study, the up-regulation of JAR1, JAZ, and WYC2 was detected in the signal transduction pathway. Meanwhile, the metabolism of α-linolenic acid (ko00592) was also involved in the browning response; α-linolenic acid is not only a precursor of both ethane (Konze and Elstner, 1978) and traumatic acid (a wound hormone associated with wounding and cell proliferation; Zimmerman and Coudron, 1979), but can also be released into the cytoplasm from plant cell membrane lipids and rapidly converted to JA or MeJA in cells (Farmer et al., 1992; Croft et al., 1993; Stark et al., 2008) (**Supplementary Figure S5**). Accordingly, it can be inferred that the JA signaling pathway is likely to work as an important long-distance wound signal transmitted from the cutting site to the shoots of *M. formosana* and mediating the browning response after cutting.

Additionally, the transient expression of PFK-1 (comp56188_c0), HMGR (comp54246_c0), and mTORC1(comp57176_c1) after cutting in *M. formosana* suggests that the AMPK signaling pathway (ko04152) may be activated during browning, which would inhibit cell growth, protein synthesis and promote autophagy (Emanuelle et al., 2015) (**Supplementary Figure S6**). We deduce that the poor growth of *M. formosana* and partial necrosis of the leaves after cutting is probably a self-protection strategy of the plant modulated by the AMPK signaling pathway to help survive the stress. However, DEGs in other pathways still deserve further research to gain a better understanding of the signal transduction responsible for browning, and the cross-talk amongst those signaling pathways also needs exploration.

### DEGs with Homology to Known Genes Involved in Secondary Metabolism during Browning

It is well known that secondary metabolites play a major role in plant adaptation and defense to adverse environments, although they have no fundamental function in the maintenance of plant life processes (Ramakrishna and Gokare, 2011). The accumulation of secondary metabolites often occurs in plants subjected to environmental stresses, and may be conducive to plant self-protection (Dixon and Paiva, 1995; Saltveit, 2000). For plants cultured *in vitro*, the species liable to browning are usually found to produce a higher content of phenolic compounds (Casteele et al., 1981; Dalal et al., 1992; Peiser et al., 1998; Ahmad et al., 2013). In this study, key enzymes functioning in the biosynthesis of phenolic compounds, such as PAL (comp66038_c0), flavonoid 3′-hydroxylase (comp36974_c0), cinnamoyl-CoA reductase (comp72476_c0), and cinnamyl alcohol dehydrogenase (comp48947_c0, comp60484_c0), all had consistently enhanced expression for a relatively long time after cutting (Balasundram et al., 2006). It seems that various phenolic compounds (tannin, lignin, flavonol, and anthocyanin) briefly accumulated in *M. formosana*, and that self-protection reactions depending on secondary metabolites were initiated after cutting. However, the production of abundant phenolic compounds is unfavorable for plant growth.

Additionally, it has been suggested that the resulting quinones and condensed tannins (the derivatives of phenolic compounds) are not only toxic to plants, but are also the main component of the brown substance at the cutting site (Laukkanen et al., 1999; Ahmad et al., 2013). Recently, the key chromophores in the browning of Iceberg lettuce were identified as yellow sesquiterpenes by a combination of multilayer countercurrent chromatography, high-performance liquid chromatography, nuclear magnetic resonance, and mass spectrometry techniques; They were named lettucenins and proposed to be a new type of secondary metabolite associated with the browning response (Mai and Glomb, 2014). In our present study, the biosynthesis of terpenoids was also detected after cutting at the transcription level, as well as polypeptides and antibiotics. In contrast, the next generation sequencing technology used in our study is an effective means to comprehensively find new types of secondary metabolites responsible for browning. However, their functions during browning still need further study and verification.

## Conclusion

This study reports a progressive and detailed characterization of the browning response in *M. formosana* after cutting and proves that the browning is not lethal. The combination of *de novo* transcriptome sequencing and DGE profiling based on Illumina sequencing technology proved to be a rapid and powerful method to explore the molecular regulation of browning. Putative DEGs involved in the stress response, signaling pathways, and secondary metabolism were highlighted and discussed to study the molecular mechanism of browning after cutting. To our knowledge, this is the first publication of transcriptome and DGE profiling for *Medinilla* without prior genome annotation. The bioinformatics data from this study provide an important resource for understanding the molecular mechanism of plant browning in *M. formosana*, and might help develop strategies to eliminate browning damage more effectively in the future.

## Author Contributions

Conceived and designed the experiments: YW, YtW, and JC. Performed the experiments: YW, KL, and XS. Analyzed the data: YW. Contributed reagents/materials/analysis tools: YW and YtW. Wrote the paper: YW.

## Conflict of Interest Statement

The authors declare that the research was conducted in the absence of any commercial or financial relationships that could be construed as a potential conflict of interest.

## Footnotes

1 http://cran.r-project.org/web/packages/pheatmap/index.html

2 http://www.genome.jp/kegg/

## Supplementary material

FIGURE S1 **Correlation analysis of DGE libraries.** Dots in the figures indicate individual tag entities. Pearson correlation coefficients (R^2^) are shown in the lower right corner of each plot. The correlation between 0 h, 4 h, and 4 days libraries are shown.

FIGURE S2 **Coefficient analysis between gene expression ratios obtained from DGE and qRT-PCR data**.

FIGURE S3 **KEGG pathway of plant hormone signal transduction**.

FIGURE S4 **KEGG pathway of plant-pathogen interaction**.

FIGURE S5 **KEGG pathway of alpha-Linolenic acid metabolism**.

FIGURE S6 **KEGG pathway of AMPK signaling**.

TABLE S1 **Annotation of unigenes from *M. formosana* transcriptome**.

TABLE S2 **DEG sub-clusters with different expression patterns**.

TABLE S3 **Primer sequences of RT-PCR analysis**.
