# Supplementary figures and images for: Characterization and Comparative Expression Profiling of Browning Response in Medinilla formosana after Cutting
Source: Front Plant Sci. 2016 Dec 22;7:1897. doi: 10.3389/fpls.2016.01897 (PMC5178855; doi:10.3389/fpls.2016.01897)

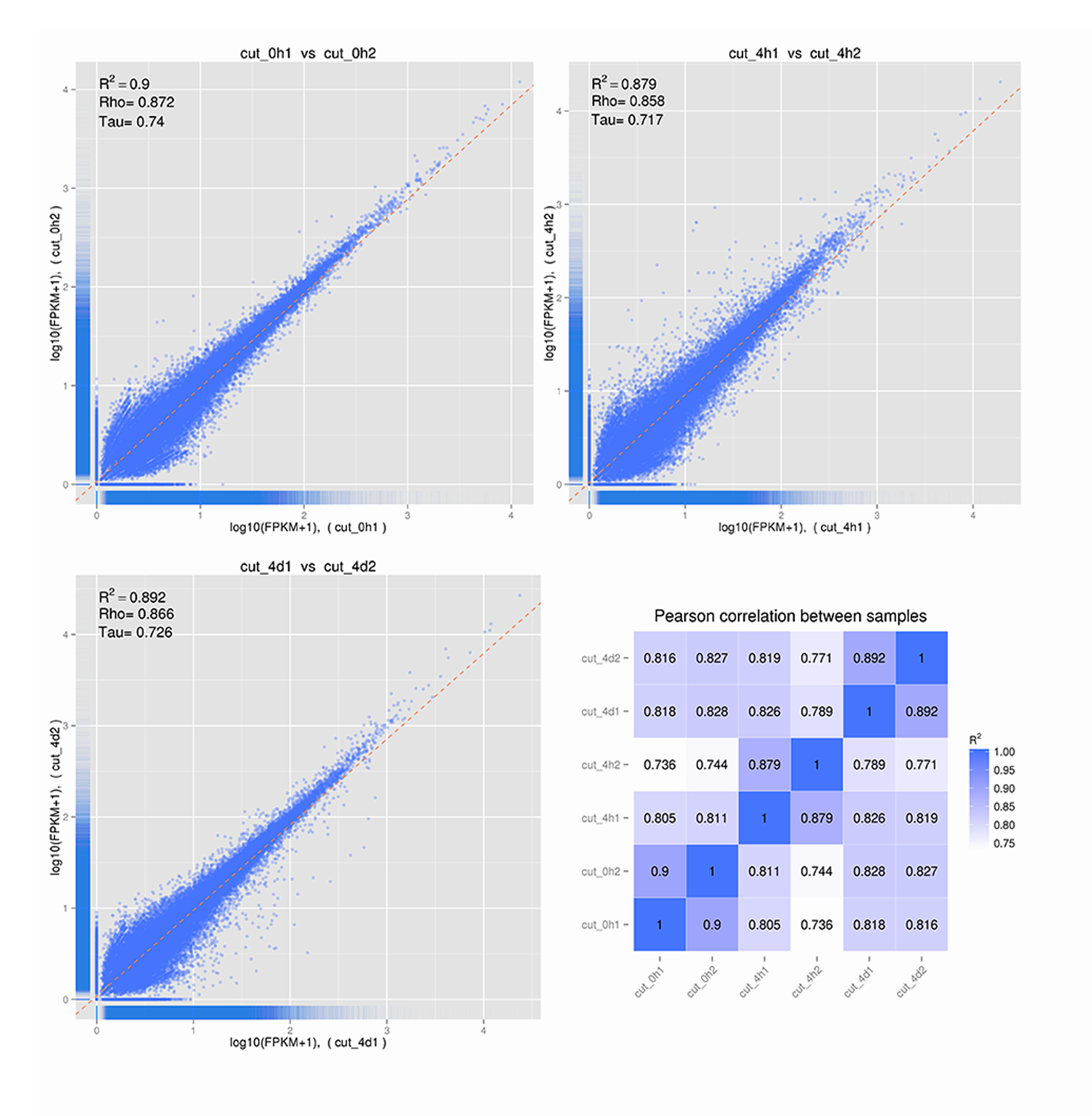

Supplement: FIGURE S1 — Correlation analysis of DGE libraries. Dots in the figures indicate individual tag entities. Pearson correlation coefficients (R2) are shown in the lower right corner of each plot. The correlation between 0 h, 4 h, and 4 days libraries are shown. [file Data_Sheet_1.zip › Data_Sheet_1/Image 1.TIF]

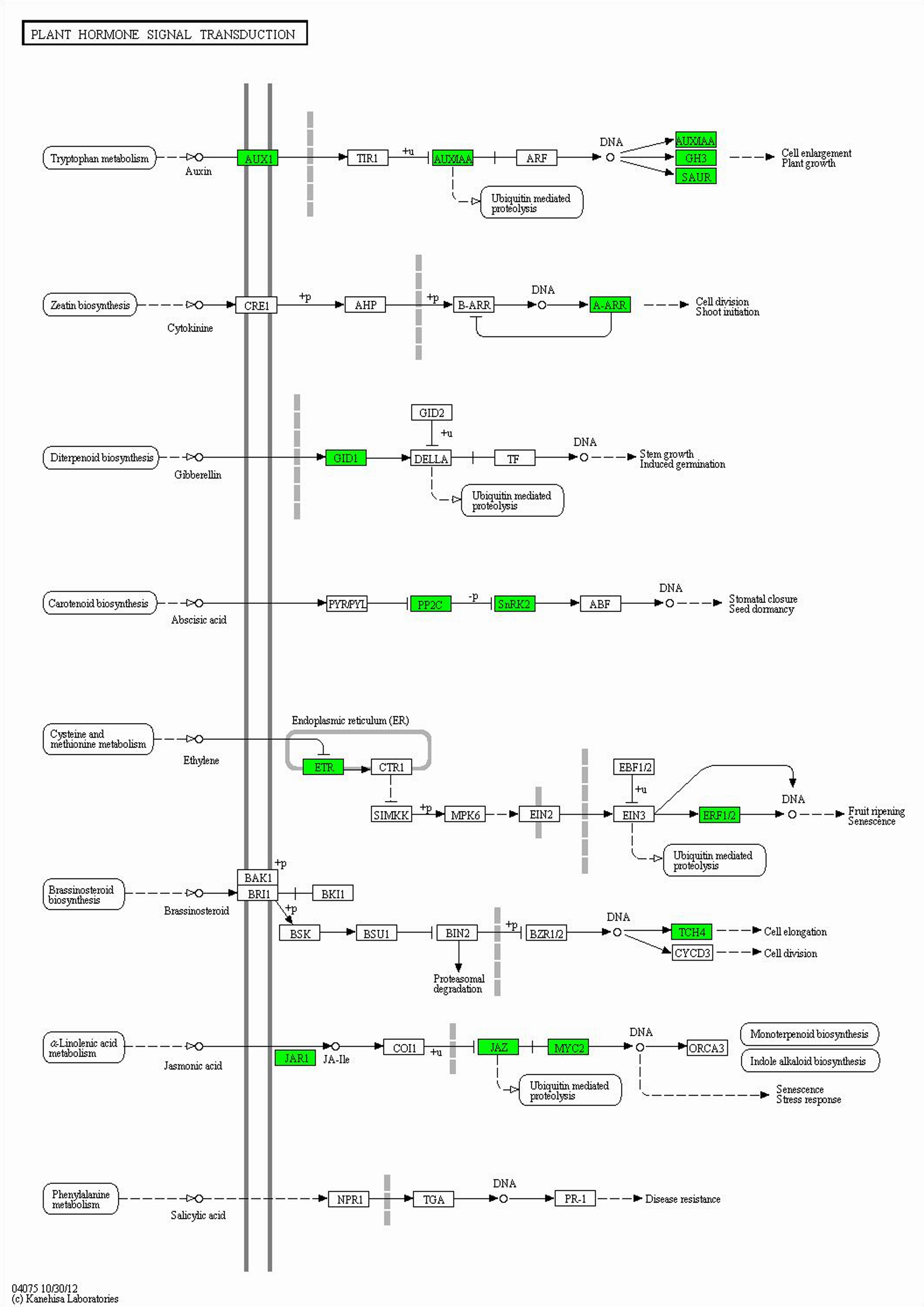

Supplement: FIGURE S1 — Correlation analysis of DGE libraries. Dots in the figures indicate individual tag entities. Pearson correlation coefficients (R2) are shown in the lower right corner of each plot. The correlation between 0 h, 4 h, and 4 days libraries are shown. [file Data_Sheet_1.zip › Data_Sheet_1/Image 2.TIF]

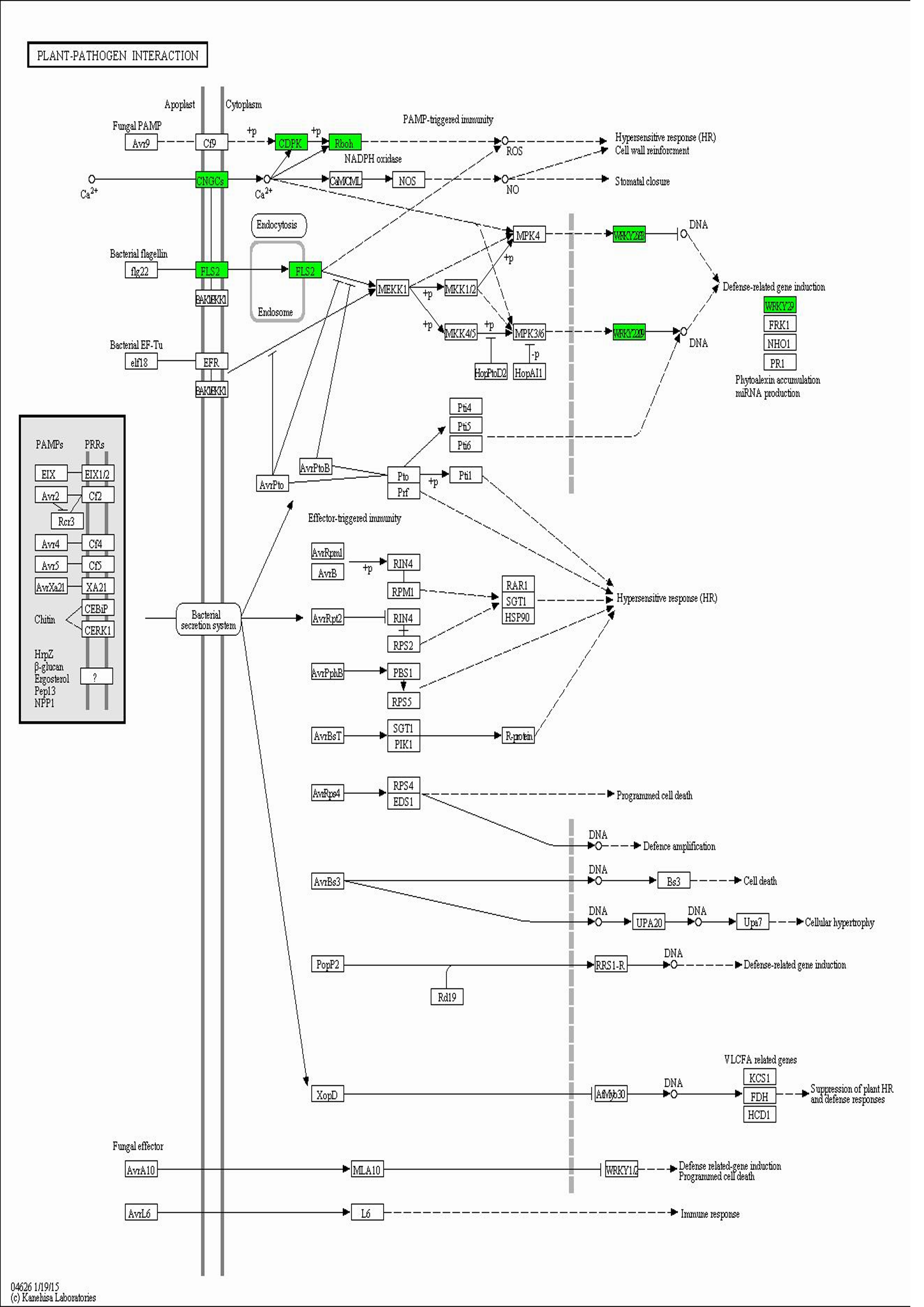

Supplement: FIGURE S1 — Correlation analysis of DGE libraries. Dots in the figures indicate individual tag entities. Pearson correlation coefficients (R2) are shown in the lower right corner of each plot. The correlation between 0 h, 4 h, and 4 days libraries are shown. [file Data_Sheet_1.zip › Data_Sheet_1/Image 3.TIF]

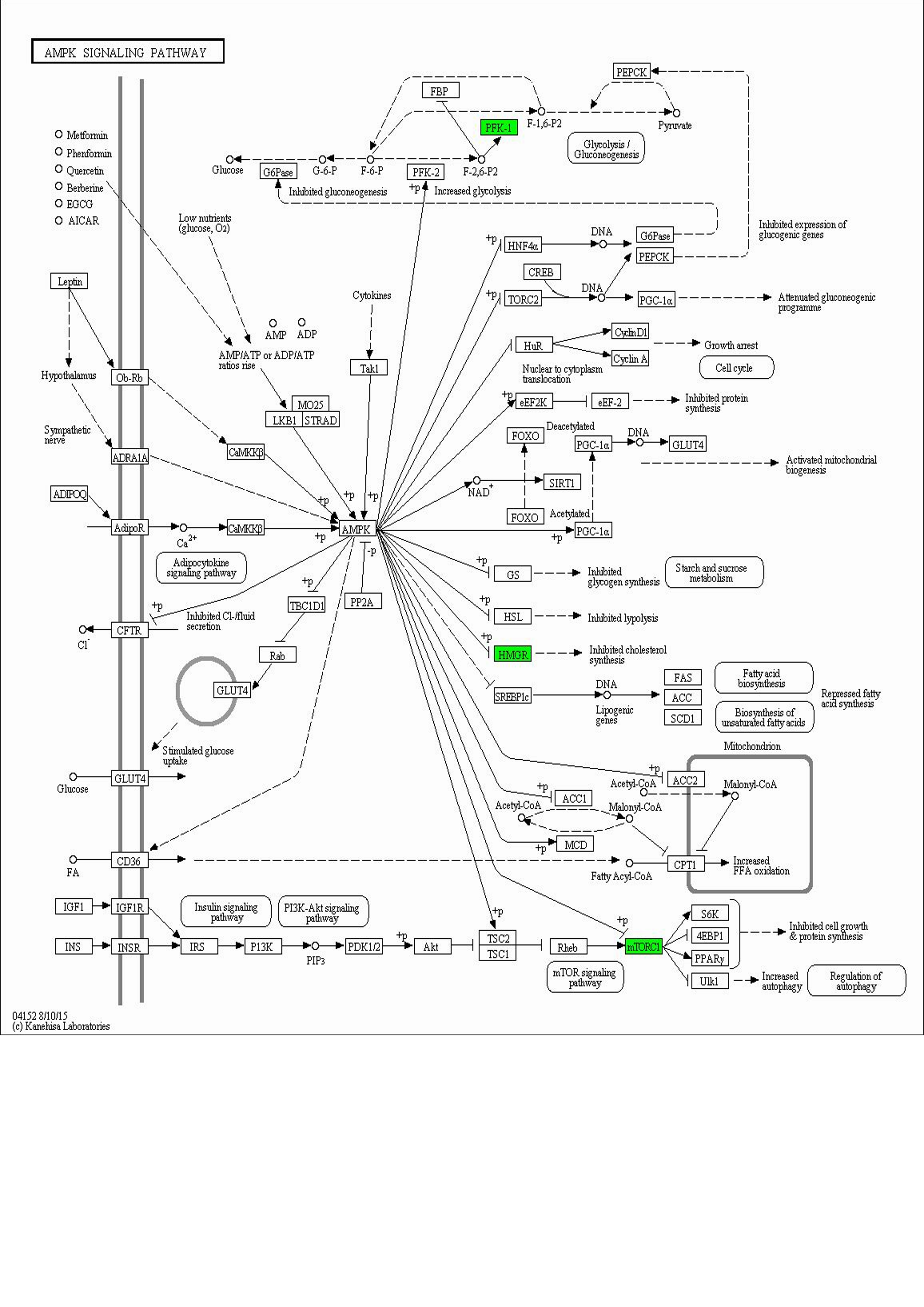

Supplement: FIGURE S1 — Correlation analysis of DGE libraries. Dots in the figures indicate individual tag entities. Pearson correlation coefficients (R2) are shown in the lower right corner of each plot. The correlation between 0 h, 4 h, and 4 days libraries are shown. [file Data_Sheet_1.zip › Data_Sheet_1/Image 4.TIF]

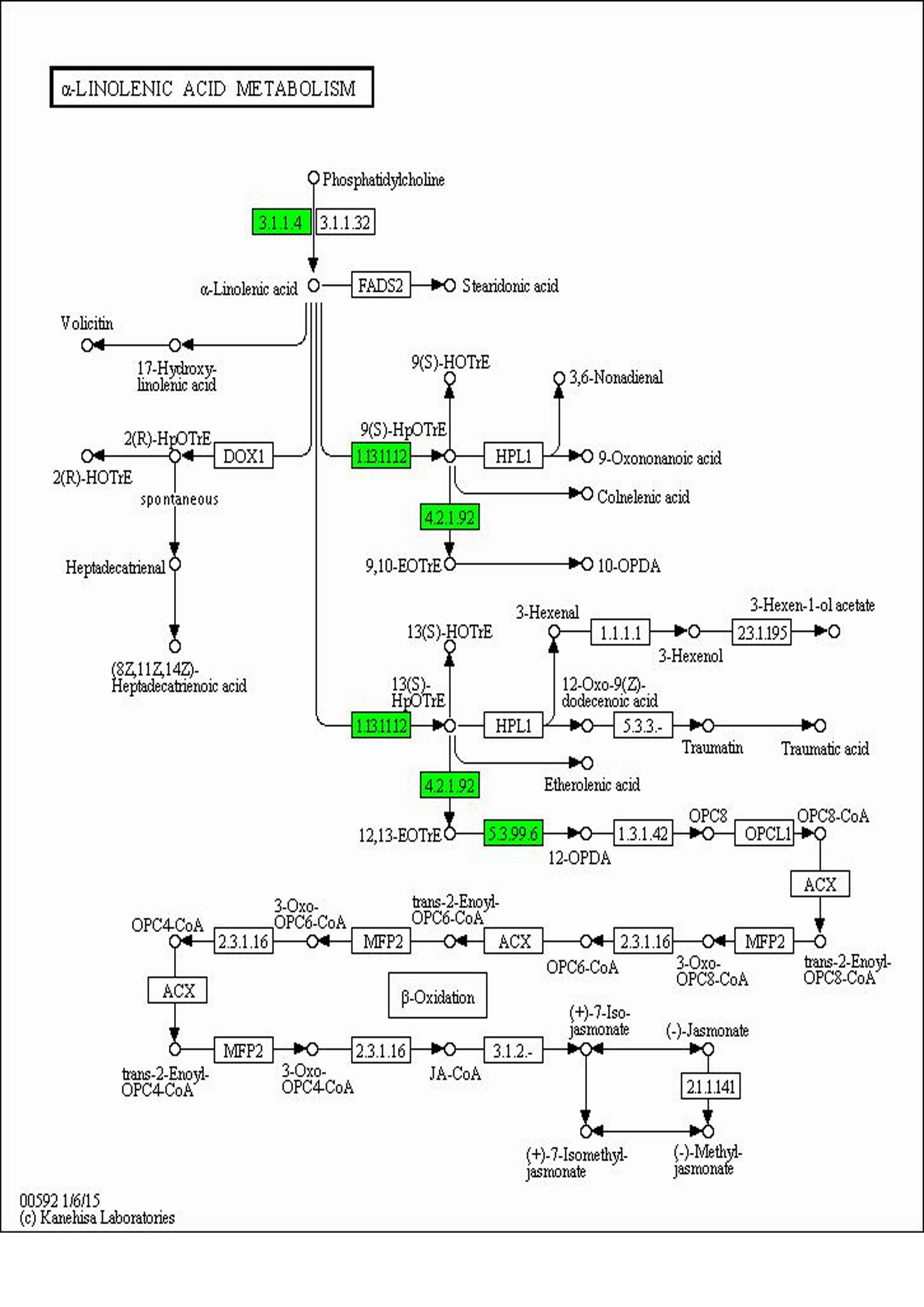

Supplement: FIGURE S1 — Correlation analysis of DGE libraries. Dots in the figures indicate individual tag entities. Pearson correlation coefficients (R2) are shown in the lower right corner of each plot. The correlation between 0 h, 4 h, and 4 days libraries are shown. [file Data_Sheet_1.zip › Data_Sheet_1/Image 5.TIF]

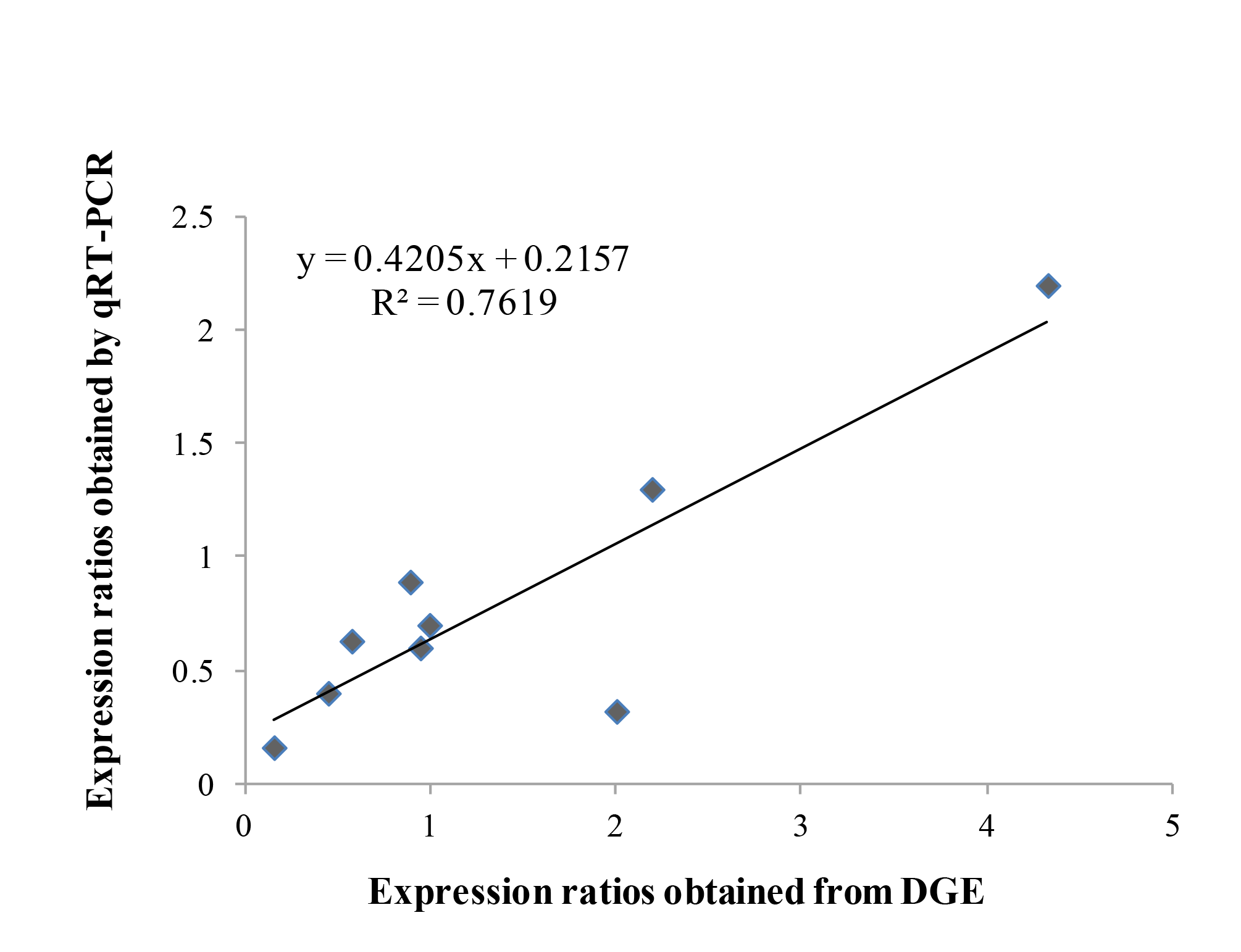

Supplement: FIGURE S1 — Correlation analysis of DGE libraries. Dots in the figures indicate individual tag entities. Pearson correlation coefficients (R2) are shown in the lower right corner of each plot. The correlation between 0 h, 4 h, and 4 days libraries are shown. [file Data_Sheet_1.zip › Data_Sheet_1/Image 6.TIF]
